# Implementation strategies: lessons learned during an e-learning intervention to improve dietary behaviors and feeding practices in early childhood education and care

**DOI:** 10.1186/s40795-024-00990-3

**Published:** 2025-01-13

**Authors:** Henrik Lian, Nina Cecilie Øverby, Frøydis Nordgård Vik, Anine Christine Medin, Natalie Garzon Osorio, Christine Helle, Tormod Bjørkkjær, Penelope Love, Harry Rutter, Mary Elizabeth Barker, Elisabet Rudjord Hillesund, Sissel Heidi Helland

**Affiliations:** 1 Centre for Lifecourse Nutrition, Department of Nutrition and Public Health, Faculty of Health and Sport Sciences, University of Agder, Postbox 422, Kristiansand, 4604 Norway; 2 Insitute for Physical Activity and Nutrition, School of Exercise and Nutrition Sciences, Deakin University, Geelong, VIC Australia; 3 Department of Social and Policy Sciences, University of Bath, Bath, UK; 4 School of Health Sciences, University of Southampton, Southampton, UK; 5 MRC Lifecourse Epidemiology Centre, University of Southampton, Southampton General Hospital, Southampton, UK

**Keywords:** Champion, Dynamic integrated evaluation model, Early childhood education and care, Expert recommendations for implementing change, Implementation strategies, Newsletters, *Nutrition Now*

## Abstract

**Background:**

Early Childhood Education and Care (ECEC) centers play an important role in fostering healthy dietary habits. The *Nutrition Now* project focusing on improving dietary habits during the first 1000 days of life. Central to the project is the implementation of an e-learning resource aimed at promoting feeding practices among staff and healthy dietary behaviours for children aged 0–3 years in ECEC. Implementing new interventions often presents challenges. This study explores ECEC staff views and experiences with selected strategies for implementing an e-learning resource in ECEC centers in a municipality in Southern Norway.

**Methods:**

The study is a part of the N*utrition Now* study, a hybrid type 1 non-randomized controlled trial. The implementation process followed the Dynamic Integrated Evaluation Model (DIEM). Implementation strategies were selected from the Expert Recommendations for Implementing Change (ERIC) project and included *identify and prepare champions*,* conduct educational meetings*,* distribute educational materials*,* create a learning collaborative*, and *remind clinicians*. ECEC teachers from participating ECEC centers in the intervention municipality were recruited as champions. Brief (5–7 min minutes), semi-structured phone interviews, covering key points, were conducted with the champions 8 times, evenly distributed over six months. The interviews were analysed using qualitative thematic analysis.

**Results:**

In total, 29 of the invited ECEC centers (53%) participated, and 260 brief interviews (88%) were conducted with champions (*n* = 37). An evaluation of the feedback from the champions suggests that the five selected implementation strategies were acceptable. Five main themes were developed by qualitative analysis: *1) Being a champion resembles what I already do. 2) Educational meetings are fine but take time. I prefer when peers share experiences. 3) Newsletters were helpful and reminded me*,* but I do not always have enough time to read. 4) Evaluations have increased my awareness*,* and we do them informally and formally. 5) The regular phone calls reminded me I could receive support and express my opinion.*

**Conclusion:**

This study’s findings suggest that several implementation strategies are acceptable for stakeholders in an ECEC e-learning healthy eating intervention. However, time constraints among champions may hinder deep engagement. These results provide valuable insights into how the selected implementation strategies may function in practice and how they are perceived and experienced by the ECECs staff.

**Trial registration:**

Trial registration on June 6, 2022: ISRCTN10694967.

**Supplementary Information:**

The online version contains supplementary material available at 10.1186/s40795-024-00990-3.

## Background

An unhealthy diet is a modifiable risk factor and a contributor to the global disease burden [1]. Early Childhood Education and Care (ECEC) centers may provide an early life arena to lay the foundations for healthy meal behaviors and positive associations with food through the lifecourse, and thus represent a potentially important setting to support early interventions to reduce the burden of non-communicable disease. The European Union defines ECEC as “*any regulated arrangement that provides education and care for children from birth to compulsory primary school age”* [2]. In Norway, all children are entitled to a place in a publicly subsidized ECEC [3] with 93% of 1–5-year-olds attending [4] consuming a significant portion of their everyday meals, totaling around 3000–4000 meals during their ECEC years [5]. The ECECs are responsible for organizing the meal times [6], presenting a significant potential for influencing children’s eating habits positively [7]. Typical ECEC staff include teachers, child and youth workers, assistants [8], and in some ECECs, chefs [5]. Fewer than 20% of ECECs have kitchen staff or use catering services. In 46% of ECECs, meals are organized around parent-packed meals, which are more common in municipal than in privately owned ECECs [9]. In Norway ECEC teachers have the responsibility of guiding and ensuring compliance with national curriculum [10], leading planning, implementation, documentation, assessment, and activity development within children’s groups [10]. ECECs usually have separate departments for different age groups: those for younger children (0–2 years old) and those for older children (3–5 years old). Other countries will have different ways of organising the ECEC and different food systems. A Cochrane review of healthy eating interventions in ECEC settings by Yoong et al. (2023), concluded that interventions may lead to small improvements in fruit consumption, and possibly also vegetable consumption, but the evidence is uncertain [11]. Yoong et al.‘s findings support the need to better understand if the interventions themselves were ineffective or due to poor implementation of them.

Implementation research aims to develop methods that promote the uptake of research findings into routine practice, thereby improving the quality of health services and care [12]. A key objective of implementation science is to enhance this process by identifying, developing, and testing implementation strategies [13]. The ERIC project [13] has compiled a comprehensive set of discrete implementation strategies, providing clear definitions to support the process. These strategies encompass various aspects, such as providing audit and feedback during the implementation, building coalitions, and conducting educational outreach visits. Such strategies are commonly evaluated using the implementation outcomes of *acceptability* (agreeable, palatable, or satisfactory), *appropriateness* (fit, relevance, or compatibility), and *feasibility* (the extent to which a new treatment or an innovation, can be successfully used) following the implementation evaluation framework by Proctor et al. (2011) [14].

Wolfenden et al. (2020), reviewed studies on implementing policies and practices for healthy eating in ECEC settings [15]. They suggest that implementation strategies likely enhance the execution of policies, practices, or programs aimed at promoting healthy eating, physical activity, and/or preventing obesity in ECEC [15]. However, the true effect may be substantially different from the reported estimated effect. Wolfenden et al. acknowledge that their findings are limited by the small number of randomized controlled trials (RCTs), small sample sizes, and the limited number of strategies evaluated, with most research conducted by a few groups and only one study originating from Europe. They recommend involving a broader range of research groups and contexts to strengthen the evidence base [15]. This includes gaining a thorough understanding of the setting, knowledge of barriers, and carefully selecting support and implementation strategies tailored to address these challenges. This underscores the need for additional data from new studies focusing on implementing healthy eating interventions in ECEC.

The current study is part of the broader *Nutrition Now* project aiming to improve staff’s feeding practices and dietary behaviours for 0–2-year-olds. The Nutrition Now study is a hybrid type 1 implementation study [16] and targets pregnant women and parents of 0–2-year-olds and two different municipal services, that care for their children: maternal health care clinics and ECEC. It builds on prior research and focuses on four effective interventions that have demonstrated promising improvements in addressing dietary challenges [17–20]. This paper has a limited perspective, focusing on the ECEC setting in the *Nutrition Now* study and the evaluation of some of the applied implementation strategies aimed to improve ECEC staff’s feeding practices and dietary behaviors for children aged 0–2 years. The ECEC part of the *Nutrition Now* project focuses on implementing an e-learning resource to improve dietary behaviors and feeding practices in Norwegian ECEC centers [16]. The e-learning resource is a website to which participants gain access to upon registration. The design of the resource is based on a prior ECEC intervention we conducted [21]. It has since been significantly reworked and customized for ECEC staff, with their involvement and support [22]. The modules of the e-learning resource targeting ECEC staff aims to influence the promotion of healthy food through four core components: 1) food sensory education once a week guided by the Sapere method [23], 2) monthly menu for hot lunch dishes twice a week, 3) pedagogical mealtime practice, and 4) ECEC-parental cooperation, all over a five-month period. The website included short videos and text on how to implement the components and provided information encouraging regular evaluation. A previous qualitative study among ECEC teachers, identified that implementation of this digital resources could be strengthened in ECEC centers by recruiting teachers to provide a leading role as champions for the intervention [22]. Champions are defined as “*individuals who dedicate themselves to supporting*,* marketing*,* and driving through an implementation*,* overcoming indifference or resistance that the intervention may provoke in an organization*” [13]. Furthermore, the results indicated that teachers are likely to need support, training, and reminders [22]. Building on these findings, the *Nutrition Now* project applies strategies that address this. The aim of this study was to explore champions’ views and experiences of selected implementation strategies used to support the implementation of an e-learning resource designed to improve dietary behaviors and feeding practices in ECEC centers.

## Methods

### Setting and design

This study is part of the *Nutrition Now* project, focusing on the ECEC sector in a control- and an intervention municipality in Southern Norway. The project targets key groups and settings crucial for child diet, including families/parents, healthcare centers, ECEC, and the municipal level. The focus of this paper is restricted to evaluating feedback from interviewed champions in ECECs in the intervention municipality. A qualitative approach with thematic analysis of interviews was chosen because it is well-suited for capturing participants’ opinions and subjective experiences [24] providing valuable user insights during the Nutrition Now implementation phase.

The implementation of the *Nutrition Now* resource was guided by the Dynamic Integrated Evaluation Model (DIEM) [25] with particular emphasis on its rapid, iterative evaluation and improvement cycles. Implementation strategies and their definitions were obtained from the ERIC project [13]. A pragmatic approach was used by the research team in the selection process to address previously identified barriers and facilitate implementation, drawing on prior experience and consultations with ECEC staff [22]. The strategies were selected from a broader list used in the *Nutrition Now* project [16] and were chosen because they can be directly linked to the champions’ tasks, with the aim of strengthening their ability to facilitate implementation. The following strategies were selected; *identify and prepare champions*,* conduct educational meetings*,* distribute educational materials*,* create a learning collaborative*, and *remind clinicians* [13]. The five strategies were covered as follows: 1) ECEC managers were advised to appoint ECEC teachers as *champions* tasked with supporting, promoting, and driving the implementation forward, overcoming any indifference or resistance that may arise within the organization due to the intervention [13]; 2) Two digital *educational meetings* were held at the 7th and 14th weeks post-implementation-startup. Each lasting forty-five minutes with ten minutes dedicated to sharing experiences. They were led by an expert in feeding practices. The purpose was to guide the creation of a collaborative support system. All stakeholders from the ECEC centers, including managers, champions, chefs, and other employees, were invited; 3) *Educational materials* included monthly digital newsletters with specific tasks for the ECEC to reinforce the core components of the digital resource (see Supplementary File 1); 4) The newsletters also included suggestions on how to collaborate effectively and methods to establish an *internal learning collaborative.* Both the newsletters and the e-learning website provided information encouraging evaluation of their mealtime practices to advance the progress of those. The themes addressed in the newsletters were adapted in a timely manner to align with the most relevant stages of the implementation timeline; 5) *Remind clinicians* was covered by the eight phone interviews per champion and newsletters, which also involved providing support. The outcomes were limited to acceptability, appropriateness, and feasibility, as described by Proctor et al. (2011) [14], to avoid overburdening interviewees, maintain a high response rate, and reduce complexity. These outcomes were considered most relevant for gaining insights into the strategies experienced by champions during the implementation process. The Template for Intervention Description and Replication (TIDieR) [26] and Consolidated Criteria for Reporting Qualitative Research (COREQ) [27] checklists guided the reporting of this study (see Supplementary File 2 and 3).

#### The recruitment of ECEC-centers and champions

Prior to implementation, the initiative was anchored with the managers at the municipality level and ECECs managers through meetings. All ECEC centers and their managers in two municipalities (intervention *n* = 55, control *n* = 40) were invited to participate. Champion recruitment was done only in the intervention municipality, via e-mail to participating ECECs managers, in which it was suggested and expected that they appoint an ECEC teacher for the champion role. The managers and champions were provided with written information and signed an electronic consent.

#### Data collection and interview characteristics

From October 2022 to April 2023, during the implementation phase, brief, semi-structured phone interviews were conducted with champions approximately every three weeks by the first author (HL), as part of the iterative evaluation cycles described by DIEM [25]. Each interview lasted 5–7 min. Interview guides consisted of questions relevant to the intervention timeline, and the questions aligned with newsletter themes tailored to each stage of the implementation process (see Table 1 and Supplementary Table 1). As part of the phone calls champions had the opportunity to ask questions and receive rapid problem-solving assistance. Eight interview rounds were conducted, and response rates were calculated based on 37 champions. In each round, champions were called up to three times before being categorized as non-responders in that round. The interviewer had no prior relationship with the participants, who knew the researcher was working on a related PhD.

**Table 1:** Overview of when different main topics were addressed in the interviews with champions

Interview number	1	2	3	4	5	6	7	8
**Timeline**	Oct.	Nov.	Dec.	Jan.	Feb.	Mar.	Mar.	Apr.
**Main topics**								
Champions’ perception of their role	x	x	x		x			
Experience with the distributed educational materials (the e-learning resource)	x				x		x	x
Experience with the distributed educational materials (newsletters)		x		x		x	x	x
Experience with collective learning (in relation to mealtime practices)		x		x		x		
Experience with the conducted educational meetings			x			x		

Questions were tailored before each of the eight interview rounds by three researchers (HL, SHH, FNV), to focus on evaluating the chosen implementation strategies. The last question of each interview was always left open to encourage reflection and gather any additional feedback. The interviewer noted responses in real-time on paper. These were typed up the following day. What HL perceived as out of topic conversations (i.e. talk about the weather etc.) was not documented. During the interviews, conducted during work hours, notes were taken, including direct quotes. This was done instead of using audio recordings and creating verbatim transcriptions; there would not have been enough time between rounds of evaluation interviews to produce and analyse verbatim transcripts. Data analysed in this study are, therefore, notes of conversations rather than verbatim transcripts.

#### Data analysis

The notes from these conversations form the data for this study. It is recognised that such data inherently contain the field workers’ interpretations of interviews and events. Thematic analysis was applied to analyze the data regarding the experiences of the participating champions [24]. The analytical approach taken was the same as that taken to verbatim transcripts [28]. The study embraced a relativist ontological stance and subjective epistemic approach, based on the understanding that reality is invariably constructed in relation to a specific frame of reference and shaped by individual experiences and insights [29, 30]. Coding and qualitative thematic analysis were guided by Braun and Clarke (2006, 2013) [24, 31], using an inductive approach. This method was selected due to its broad applicability, clear structure, flexibility, recognition and utilization in the research field [24]. The steps in coding and identifying themes that address the research questions are presented in Table 2 with details of how the process was performed. Three authors participated in the analysis, each with different background and expertise. HL is an experienced clinical nutritionist and a PhD-student. SHH is an ECEC teacher and chef, and NCØ is a public health nutritionist. Both SHH and NCØ are experienced researchers and have contributed to the previous studies that laid the foundation for *Nutrition Now*. Coding was conducted using NVivo 12 software. Finally, the results were translated into English by HL and reviewed for language accuracy by NCØ. To differentiate between individual champions when citing verbatim notes, each was allocated a distinct number within the range of 1 to 37, for example, indicated as (C13).

**Table 2:** Phases of the study ‘s thematic analysis (adapted from Braun & Clarke, 2006)

Phase		Thematic analysis process
**1**	**Familiarizing with data**	While the project was ongoing, four of the authors (HL, NCØ, FNV, SHH) participated in regular meetings, familiarising themselves with the data in a timely manner as it was gathered. After data collection was completed, all data was independently read by three researchers (HL, NCØ, SHH) to obtain an overview and insight.
**2**	**Generating initial codes**	Using an inductive approach, one researcher (HL) started an initial complete data coding process by identifying and categorising recurring terms as codes.
**3**	**Searching for themes**	The codes were then read by the three researchers (HL, NCØ, SHH), discussed and organized into preliminary themes.
**4**	**Reviewing themes**	The preliminary themes were then either kept as candidate themes or discarded. Candidate themes were broken down to the original data to capture issues and then regrouped according to similar semantic content to generate main themes, and subthemes.
**5**	**Defining and naming themes**	The semantic content was discussed to generate clear names for each of the 5 main themes and their 2–3 subthemes.

## Results

Among the 29 participating ECECs, 20 were privately run, and nine municipally operated. The 37 champions recruited represented 36 departments, comprising 29 ECEC teachers, four ECEC managers, three with food responsibilities, and one chef, with two of them being male. With one exception, only one champion was recruited per ECEC department. In sum, 260 brief interviews were conducted out of 296 possible, resulting in a response rate of 88%, as shown in Table 3. There were 3 dropouts (8%). These champions were excluded from the interview rounds 4, 7, and 8 due to non-responsiveness, completed involvement, and leaving the ECEC, respectively.

**Table 3:** Number of champions reached and response rate for each interview round

Interview round	1	2	3	4	5	6	7	8
Number of champions reachable	37	37	37	36^a^	36	36	35^b^	34^c^
Number of champions reached	33	34	33	34	34	33	31	28
Response rate^d^ (%)	89	92	89	92	92	92	89	76

^a^A champion was excluded from further phone calls due to non-response

^b^A second champion was no longer contacted after expressing a personal decision to conclude their own role in the *Nutrition Now* project

^c^A third champion was excluded from the study as they stopped working in the ECEC center. Total dropouts 3 (8%)

^d^All response rates are estimated from 37 champions

**Fig. 1:**
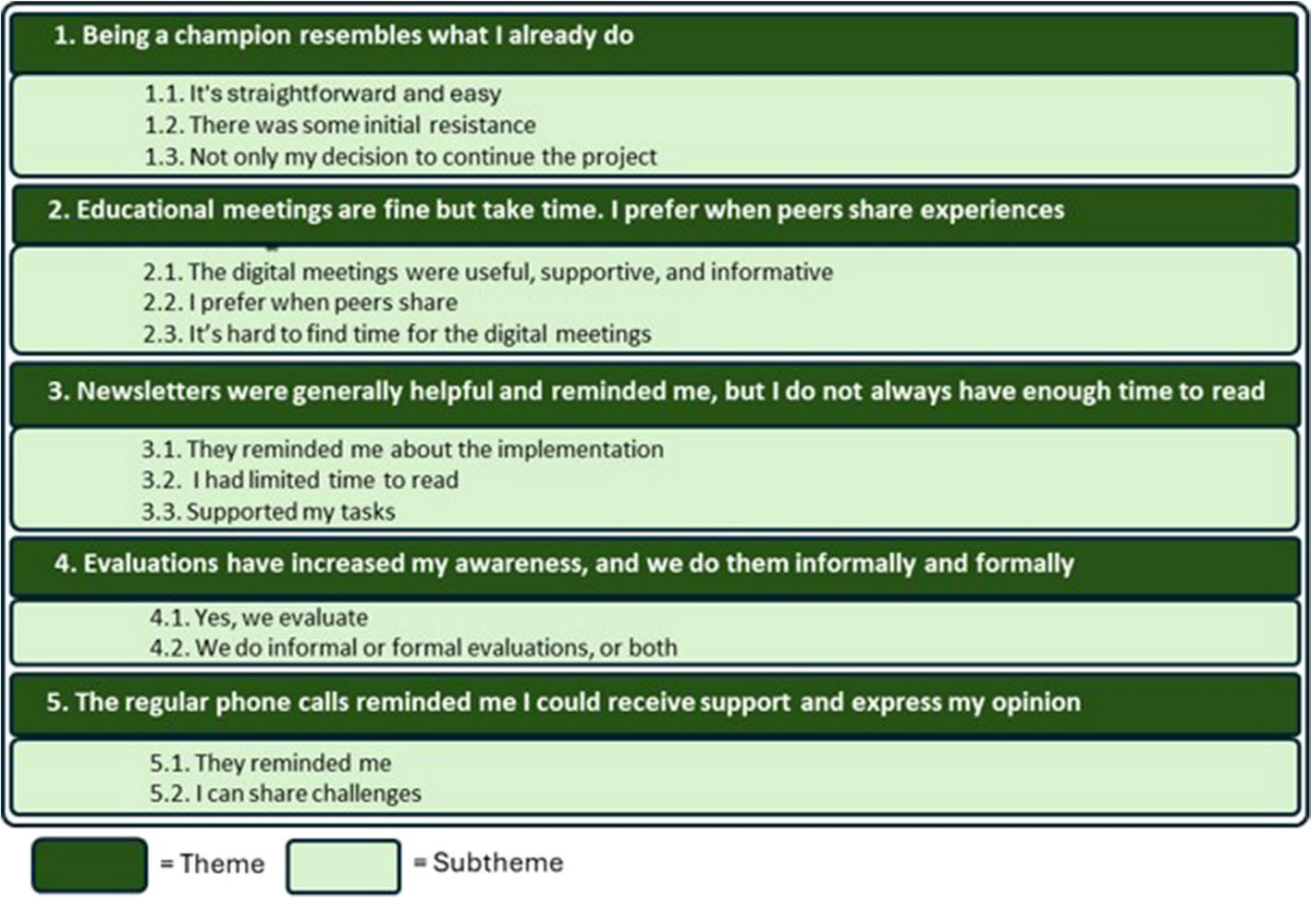
Summary of themes and subthemes presenting champions' experiences with implementing the e-learning resource *Nutrition Now*

 The findings describe the general experience of ECEC champions in their designated role and their feedback on the five selected implementation strategies for implementing the healthy eating e-learning resource, *Nutrition Now*. Champions’ responses were organized in five themes each with two to three subthemes. See Fig. 1 for summary of themes and subthemes.

1. *Being a champion resembles what I already do.* During four of the eight interviews (see Table 1), champions were invited to elaborate on their role as implementers of a digital resource, covering the strategy of identifying and preparing champions.
  1.1 *It’s straightforward and easy.* Most of the champions described the role as a champion as well-known and easy to carry out. They perceived the responsibility of being a champion as similar to their daily role as ECEC teachers. One champion answered: *A completely fine [role]. No hassle. [I’m] used to lead in the ECEC center* (C13). Already in the initial interviews the champions said: *It goes well.* Over time their responses showed that it worked even better, by responding: *It’s going very well.* About halfway into the intervention period four of the champions said that the implementation ran automatically. One said: *We’re in it now. It’s on autopilot* (C2).
    1.2 *There was some initial resistance.* A few of the champions stated that there was some skepticism from coworkers initially, who thought the intervention would be too resource demanding and time consuming. One said during the first interview: *It’s going fine. The others are positive. [But] some are skeptical due to the time commitment* (C6). But the skepticism seemed to decline after some use of the *Nutrition Now* resource, and in a later interview a champion said: *There was some skepticism initially due to resource usage in the kitchen. It [the attitude] has turned around now…. They [coworkers] are very positive after seeing that it works* (C1).
      1.3 *Not only my decision to continue the project.* There was some uncertainty among the champions regarding the continuation of the project after the project period. It seems that the decision lay with their leaders rather than with the champions themselves. One champion mentioned: *We are having a staff meeting on Tuesday. We will discuss it then* (C6). Another expressed uncertainty, stating: *[I’m] unsure about what the manager wants’ (C32).*
2. *Educational meetings are fine but take time. I prefer when peers share experiences.* The two digital educational meetings (Table 4) were conducted as information and lecture sessions, and after each meeting, champions were asked about their experience. These meetings covered the implementation strategies of conducting educational meetings and creating a learning collaborative.
  2.1 *The digital meetings were generally useful, supportive, and informative.* The champions described the meetings as a positive opportunity to apprehend information and receive support for implementing the digital resource. One champion expressed: *The meeting was actually fine. It was informative* (C9). Several champions found the meetings useful with statements like, *I think it’s useful*, (C6) and *It was very nice. Consistent with what we have learned [from the e-learning resource]. Feel free to arrange a new meeting* (C29). Some mentioned that the digital meetings served as a helpful repetition of the core components. One champion said: *Yes*,* it was a nice refresher. [I] knew a lot [of the information] from before. Useful with repetition* (C27). However, one champion who did not participate expressed uncertainty about the usefulness of digital meetings: *Forgot it [the digital meeting]. A lot of illness [in the department]. Uncertain if it’s useful. Managed fine without it* (C21). Another said: *Didn’t get much out of it. I’ve been working on this and have good routines. Have read through it on the website* (C15).
  2.2 *I prefer when peers share.* After the digital educational meetings, some champions expressed a desire to learn from other ECECs’ approaches to the core components. Ten minutes of the forty-five minutes per meeting were dedicated to sharing experiences, with several champions expressing appreciation for the opportunity. One said: *Absolutely useful to talk to the others involved in the project* (C18). Another champion said: …*nice with a digital meeting where we could talk to other ECECs. I wish for more meetings with other ECECs where we can share tips and [discuss] what we do* (C16).
  2.3 *It’s hard to find time for the digital meetings.* The champions attendance dropped from the first to the second digital meeting, see Table 4. Some of the champions said that lack of time due to other work tasks was the main reason for this. Examples of such tasks were extra efforts needed when new children started in ECEC, or that they did not have enough staffing due to illness. One champion said: *I didn’t have the opportunity due to the enrolling of new children* (32). Another said: *Was not possible because the manager was on sick leave* (22).
3. *Newsletters were helpful and reminded me, but I do not always have enough time to read.* Champions received newsletters monthly via e-mail and were asked about their experiences with these. This aimed to cover the strategies of distribute educational materials, create a learning collaborative, and remind clinicians.
  3.1 *They reminded me about the implementation.* Several champions mentioned that the newsletters served as reminders about the implementation. Nine responded: *Great with newsletters as reminders* (C7). One champion explained the importance of the reminders as *Important to receive reminders to have arguments for continuing with Nutrition Now* (C22).
  3.2 *I had limited time to read.* Quite often the champions expressed that they lacked time to read or implement the advice from the newsletters. The most common reasons were colleagues on sick leave or having to prioritize other tasks. Additionally, not all champions had enough office hours to read e-mails regularly. One expressed: *With the limited planning time we have*,* there isn’t always time to go through them [newsletters]* (C8). Some of the champions didn’t seem to have read the newsletters, or noticed it in the e-mail-box, and one answered: *I can’t remember the newsletter* (C3).
  3.3 *Supported my tasks.* Several champions expressed that they perceived the newsletters as helpful and awareness-raising. Others mentioned that they prompted self-reflection. One champion stated: *Tips on evaluation have been very enlightening* (C5). Additionally, some champions also utilized the newsletters to disseminate information to other staff members. When asked about initiating development processes among the staff, one champion replied on the information received about evaluation and fostering a collaborative learning environment through the newsletters: *Yes*,* absolutely. Definitely. Easier to get them [other staff] on board. Easier to get the others to understand* (C18). Another champion described the information as: *Very convenient for involvement. I believe that the others find it useful* (C2). However, not all champions perceived the newsletters as useful. One explained that she/he received enough information from the startup-e-mails and the *Nutrition Now* e-learning resource: *I haven’t really looked closely at it [newsletters] now. I immersed myself in Nutrition Now from the beginning* (C20).
4. *Evaluations have increased my awareness, and we do them informally and formally.* Through the e-learning resource and the newsletters, the champions were encouraged to evaluate the implementation process. They were asked to reflect on their use of internal evaluation of the four intervention core components, as part of the strategy to foster a collaborative learning environment among the staff.
  4.1 *Yes, we evaluate.* The majority said they evaluated their practices. One champion said: *Yes*,* constantly evaluating. Looking at what works. We are constantly talking about it together* (C14). Several champions evaluated only specific components of the intervention, such as the menus, feeding practices or food sensory education sessions. However, none mentioned evaluating parental collaboration. Instructions provided through the digital resource, newsletters, and educational meetings, seemed to raise awareness among the champions. For example, *one champion said: Yes*,* in a way. [I am] more conscious* (C1). A few champions noted that evaluation was not a common practice, but they acknowledged it and expressed intentions to start. One champion said: *No*,* I’ve thought about it. Good idea to evaluate. I will bring it up at the staff meeting. But we talk together about it [Nutrition Now]* (C2). Some champions also cited time constraints hindering evaluation efforts.
  4.2 *We do informal or formal evaluations, or both.* Champions outlined three main approaches for evaluation discussions, including informal, formal, or combined evaluations. Many noted that the implementation evaluation was formally organized but held at different frequencies, ranging from weekly to monthly. One champion expressed it like this: *Goals and methods are evaluated every month as part of the monthly plan* (C12). Some champions emphasized close collaboration among staff, with daily, informal, sporadic talks incorporating evaluation: *We are together all the time as a team. We don’t need meetings. We have small conversations every day* (C27). Lastly, other champions said that they combined both approaches: *[We] work closely together*,* … Don’t need to sit down. [We] are close*,* easy to communicate. [We have] departmental meeting every 14 days and have talked about it every time* (C13).
5. *The regular phone calls reminded me I could receive support and express my opinion.* The intention of the phone calls, aside from gathering information for the researchers, was to cover the strategy of remind clinicians, which also involved providing support to the champions when needed. The champions were not specifically asked about their perception of the phone calls. However, relevant information regarding this aspect emerged during other inquiries. Champions had the opportunity to have a direct dialogue with the researchers/interviewer, allowing them to ask questions and receive rapid problem-solving assistance. Through the dialog with the interviewer the champion received immediate support and was encouraged to adapt the menu or switch to less time-consuming recipes.
  5.1 *They reminded me.* Some champions found that regular phone calls served as a useful reminder and an important follow-up for the implementation. One champion expressed: *It is important to get regular reminders to provide justification to continue with Nutrition Now* (C22). Another champion said: *[I] feel that we’ve introduced something new to the ECEC. It’s important for us*,* the children*,* and the parents. It’s been a lot of fun. Follow-up is important (*C20). In the sixth interview round, one champion even stated that the calls were crucial for completing the intervention: *I think that the meetings and the fact that you call mean that we get it done*,* that it does not fizzle out. It may not be useful here and now*,* but it is important for the implementation* (C26).
  5.2 *I can share challenges.* Direct communication with champions provided the research group of the project with valuable information. For example, monitoring the intervention’s progression over time and identifying current facilitators and barriers revealed new insights. Early in the implementation, it was found that a few ECECs had not started the intervention. One champion said: *The department has not had the opportunity to review it [the Nutrition Now e-learning resource]. Due to staffing constraints*,* …* (C8). Another identified barrier was expressed as: *We must have a meeting with the manager to get started properly. [We] have not done that yet* (C4). In the second phone call three weeks later, all the champions confirmed that their ECEC center was practicing some or all core components included in the digital resource. This information, both regarding the experienced barriers and their resolution, provided the research group with an overview of the process, and the opportunity to act timeously if internal barriers persisted.

**Table 4:** Educational meetings attendance (number attending (%))

Participants	First meeting at week 7	Second meeting at week 14
ECEC centers^a^	19 (66%)	14 (48%)
ECEC departments	23 (64%)	16 (44%)
Champions	23 (62%)	12 (32%)

^a^*ECEC* Early Childhood Education and Care

An overall summary of how the implementation strategies were experienced regarding implementation outcomes acceptability, appropriateness and feasibility are presented in Table 5.

**Table 5:** The implementation strategies and outcomes as interpreted by researchers from champions’ responses

Strategies^a^	Acceptability	Appropriateness	Feasibility
Identify and prepare champions	Highly acceptable	Appropriate	Feasible
Conduct educational meetings	Acceptable	-	-
Distribute educational materials	Acceptable	Appropriate	-
Create a learning collaborative	Acceptable	-	-
Remind clinicians	Highly acceptable	Appropriate	Feasible

^a^Strategies and their definitions were obtained from Powell et al. (2015), and outcomes following the ERIC project by Powell et al. (2011). (–) Barriers such as limited time, available personnel and reduction in attendance rates at the second educational meeting hindered the appropriateness and feasibility of these strategies.

## Discussion

The current study explored the experiences and views of ECEC teachers/champions regarding five implementation strategies employed during the implementation of a healthy eating e-learning resource within an ECEC setting. An iterative approach was applied for evaluation.

The role of being a champion suited ECEC teachers well due to their accustomed leadership roles, but they were not in a position to decide whether the implementation process should continue beyond the project period in the ECEC centers. The findings offer valuable insights into how the selected strategies function in practice and are perceived by the target users. The lessons learned further illuminate the practical application of these strategies, highlighting their real-world relevance. The strategies seemed to serve as effective reminders for champions, and many reported that the content was useful. However, there were barriers such as limited time and personnel available for full utilization of the strategies. The discussion of the findings will explore certain aspects of the chosen implementation strategies, aligning with the ERIC taxonomy [13]. The discussion further focuses on relating the results to the standardized implementation outcomes *acceptability*,* appropriateness*, and *feasibility* following Proctor et al. (2011) [14].

Valuable lessons learnt from the strategy *identify and prepare champions* is that champions played a crucial and positive role in the implementation of the e-learning resource in ECEC. The champions reported that the role fitted them well and was in line with their current responsibilities. It seems reasonable that a high level of education and familiarity with being a leader [10] made ECEC teachers especially suitable for the role of ‘champion’. In recent years, a notion that champions play a pivotal role in ensuring the effectiveness of healthcare-related implementation has obtained widespread acceptance [32]. The use of champions has also been related to increased use of best practices and programs [33]. These experiences are supported by our findings. However, the findings both contrast with and align with those of Barnes et al. (2021), in a comparable feasibility study of a web-based implementation intervention to improve child dietary intake in ECECs. They experienced a low uptake of the strategy to identify and prepare a center champion but high *acceptability among those who selected a champion* [34]. Barnes et al. suggest that different organizational structure could explain their low uptake, which was seen among the smaller settings [34]. We found no such differences in uptake in our study. Based on feedback from the champions in our study, the strategy to identify and prepare champions is proposed to be highly *acceptable*,* appropriate*, and *feasible* within the ECEC setting. Furthermore, based on our findings we suggest that this strategy can potentially be applicable to implementation research projects in other fields. However, one should keep in mind the differences regarding organizational structure as commented by Barnes et al. [34].

A few of the champions experienced some resistance initially from staff who thought the project would be too time consuming, however, this diminished over time with familiarity and some use of the *Nutrition Now* intervention. Limited time for preparation may have contributed to the initial resistance from colleagues [35]. Champions in our study were given only two weeks to prepare coworkers for the specific assigned tasks. Providing earlier access to the digital resource might have been helpful for the champions. Other factors, such as general resistance to change, may also be relevant. Wanberg et al. (2000), have suggested that more information about a change, participation, change-related self-efficacy can lead to increased openness to change [36]. This highlights the importance of allocating time for the champion or manager to prepare their colleagues for upcoming changes. Ross et al. (2016), further support this, recommending that champions should be included as early as possible in the implementation process [37].

At the end of the planned five-month implementation period, many champions could not confirm whether their ECEC center would continue to use the intervention. The promotion of sustained use was only mentioned in the e-learning resource and briefly in the final newsletter. In hindsight, it could have been beneficial to mention this during the educational meetings and interviews, and to suggest dialogues with ECEC leaders to promote further use of the intervention. Additionally, the results showed that decisions related to sustainability [38] were beyond the authority of the teachers but rested with the ECEC managers, who make final decisions. There seems to be a need for exploring additional and alternative implementation strategies to encourage sustainability.

During the exploration of the strategy to *conduct educational meetings*, valuable insights were uncovered. Champions expressed a desire for more opportunities to share experiences with peers during these meetings, which suggests that the ERIC strategy *promote network weaving* should be explored in future studies [13]. This aligns with Rogers et al. (2020), who suggest that peer support, akin to coaching, aiding practitioners in refining and applying professional learning and development, appears effective [39]. Investigating peer-to-peer support among different ECEC centers to promote network weaving is therefore suggested in similar settings. Our findings support holding two digital meetings over five months, which originally was decided in dialog with local stakeholders prior to the intervention. However, the reduction in attendance rates suggest long-term *feasibility* challenges due to logistical issues at ECECs.

From the strategy of *distributing educational materials* via monthly digital newsletters to champions, it was learned that newsletters were generally perceived as helpful for raising awareness and serving as reminders of the project. This aligns with findings from Finch et al. (2019), in an implementation of healthy eating policies and practices in ECEC settings, who reported that most participants found newsletters useful, although preferences regarding frequency varied [40]. Similarly, Jones et al. (2015), found that bimonthly newsletters were *acceptable* to around 60% of participants in their ECEC healthy eating implementation [41]. However, challenges such as time constraints or limited opportunities led to inconsistent readership among champions in the current study. This contrasts with findings from other studies reporting higher readership rates [42, 43]. These differences highlight the importance of considering contextual factors and preferences when designing and using newsletters as an implementation strategy. Our results emphasize the need to develop strategies to overcome readership barriers, such as lack of time. Further research into strategies to enhance newsletter *acceptability* and *feasibility* could offer valuable insights for future implementations. In summary, it appears that *educational materials*,* educational meetings*, and *regular interviews* served as reminders for the e-learning resource and were *acceptable* for champions in an ECEC setting. Due to their stated relevance, newsletters, as part of the strategy to *distribute educational materials*, were also considered *appropriate*.

The strategy of *creating a learning collaborative* was explored. Champions were encouraged through newsletters and educational meetings to allocate time to evaluate their work and learn from each other to improve the implementation of the e-learning resource. Their tasks included ongoing internal evaluations and maintaining focus on enhancing the implementation process. Some champions noted that the guidance highlighted the importance of regular evaluation, which varied across ECEC centers from structured meetings to informal conversations or a mix of both. These findings show that ECEC centers adapt recommendations for evaluation to their organizational structures, capacity, or preferences. Feedback from champions didn’t confirm the creation of a learning collaborative as defined by Powell et al. (2015) [13], but the iterative interviews suggest partial success. Time constraints and inconsistent readership of newsletters raise doubts regarding the *feasibility* of implementing this strategy solely through newsletters and online educational meetings.

The iterative interviews with the champions served to monitor the implementation processes and outcomes for quality assurance, using staff and champions’ feedback to increase implementation efforts. Some champions viewed the regular, short phone interviews as reminders for implementation and support. This aligns with Gruß et al. (2020), who found that the phone check-ins served as reminders and positively influenced implementation activities [44]. Finch et al. (2012), reported that 49% of service managers found support calls very useful in helping to implement a physical activity program in ECECs [43], and participants in their healthy eating ECEC implementation study also found calls helpful, motivating, and acceptable [40]. Similarly, Jones et al. (2015), and Barnes et al. (2021) found that most participants viewed telephone support as *acceptable* [34, 41]. These findings support our findings, and the high interview response rate of 88% over time, suggest that monthly, short phone interviews are highly *acceptable*,* appropriate*, and *feasible* for ECEC champions during an implementation process. The brief, conveniently scheduled calls likely contributed to the high response rate. Further research is warranted regarding the effectiveness of the use of regular, short phone interviews to support intervention implementation within time-poor settings. To some extent, our findings align with a process evaluation of an intervention in family childcare homes in Massachusetts (USA) aimed at improving diet quality. This evaluation revealed high participation in monthly support calls that included brief motivational interviewing and newsletters, but low participation in group meetings [45].

Although this is a qualitative study, we believe our findings are in line with those of Wolfenden et al. (2020), who found in their review that “*current research suggests implementation strategies*,* to improve the implementation (or correct undertaking) of policies*,* likely improve the implementation of practices*,* or programs by childcare services*” [15].

To summarize our findings, we found that the strategies to identify and prepare champions, conduct educational meetings, distribute educational materials, create a learning collaborative, and remind clinicians were acceptable in an ECEC setting. Additionally, the strategies to identify and prepare champions and conduct regular short interviews (as reminders) were deemed both appropriate and feasible. However, barriers such as limited time and available personnel hindered the feasibility of distributing educational materials and impacted the appropriateness and feasibility of creating a learning collaborative. Additionally, reduced attendance rates at a second educational meeting affected the appropriateness and feasibility of conducting these meetings.

### Strengths and limitations

One strength of this study was the use of qualitative methods to contextualize the role of champions and the selected implementation strategies in driving the digital healthy eating resource in ECEC. Another strength was the involvement of municipal- and ECEC management. The research group also had prior knowledge of barriers, facilitators, and practical needs in the ECEC environment from previous studies on diet quality and mealtime environment [16, 22]. High response rates and consistent interviewing by one person allowed for continuous tracking of implementation-related changes over time. This approach may also have facilitated the development of a trusting relationship with champions, potentially leading to more substantial responses as they grew accustomed to the interviewer and the interview format. However, relying on local implementation personnel may have introduced social desirability bias, where responses could have been influenced by the desire to present positively or meet interviewer expectations. One weakness is that some authors were part of both the *Nutrition Now* project’s inception and the development of the digital healthy eating resource. This could introduce biases like partiality and limited diversity of perspectives, potentially affecting the objectivity of reporting. However, the interviewer’s lack of involvement in the development may have mitigated these biases. Furthermore, interview notes, including direct quotations, were taken during the interviews instead of audio-recording with verbatim transcripts. Although the interviewer aimed to take accurate notes which contributed to not capturing all details in the interview and being less accurate compared to verbatim transcripts, which may undermine the study’s trustworthiness. This study is in effect a re-analysis of the interviews which therefore limits the interpretation of meaning in participants’ accounts [46]. Despite the brief responses due to the short duration of each interview, conducting them as a series with many participants over time still provided valuable insights into the implementation process.

## Conclusion

We found the following implementation strategies to be acceptable within an ECEC setting when implementing an e-learning resource to improve staffs feeding practices and children’s dietary behaviors: *identify and prepare champions and remind clinicians*, which were highly acceptable, as well as *conduct educational meetings*,* distribute educational materials*, and *create a learning collaborative*, which were considered acceptable. However, time constraints among champions seem to hinder feasibility of deep engagement in tasks provided by online educational meetings and educational material distributed as newsletters. Our study adds to the limited evidence base on important and useful implementation strategies for dietary interventions in ECEC. This knowledge is valuable for others as it is grounded in real-world experiences from end users, providing practical relevance. The findings increase the likelihood that the strategies can be applied in similar settings with minimal need for extensive adaptation, enhancing their validity for implementation. While context-dependent, the results also contribute significantly to the broader discussion on ways of implementing healthy eating e-learning resources in ECEC settings. These results provide important insights to inform the scale up of the current and similar interventions. We recommend that further implementation studies should be conducted to explore effective adoption and sustained impact of specific implementation strategies.

## Supplementary Information

Supplementary Material 1. Newsletters 1–6.

Supplementary Material 2. The Template for Intervention Description and Replication (TIDieR).

Supplementary Material 3. Consolidated Criteria for Reporting Qualitative Research (COREQ).

Supplementary Material 4: Supplementary Table 1. Interview guides.

## Data Availability

The dataset supporting the conclusions of this article is provided within the article itself. While the full datasets utilized in the present study are not publicly accessible to ensure the privacy and confidentiality of our research participants, they can be obtained in anonymized format from the corresponding author upon reasonable request.

## Abbreviations

COREQ: Consolidated Criteria for Reporting Qualitative Research
DIEM: The Dynamic Integrated Evaluation Model
ECEC: Early childhood education and care
ES: Educational Supervisor
ERIC: The Expert Recommendations for Implementing Change
TIDieR: The Template for Intervention Description and Replication
