# Supplementary material for: Implementation strategies: lessons learned during an e-learning intervention to improve dietary behaviors and feeding practices in early childhood education and care
Source: BMC Nutr. 2025 Jan 13;11:7. doi: 10.1186/s40795-024-00990-3 (PMC11726949; doi:10.1186/s40795-024-00990-3)
Supplement: Supplementary file 1 — Supplementary Material 1. Newsletters 1–6. [file 40795_2024_990_MOESM1_ESM.pdf]

First Newsletter – (Week 41)

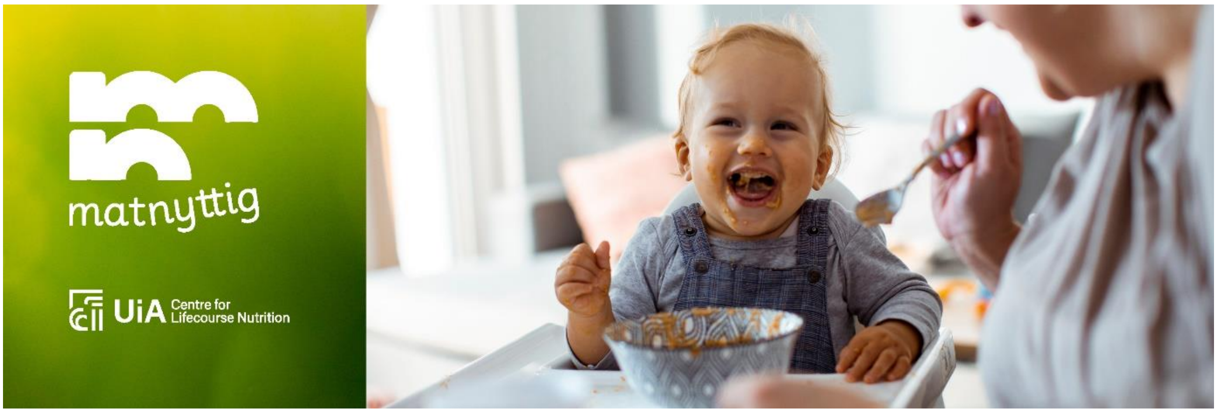

Welcome

Getting Started with Nutrition Now

Plan Your Menu and Gatherings Begin by selecting Monthly Menu 1 from the list, and inform your colleagues about the menu, assign tasks, and create a shopping list. Serve the children the two lunch dishes two days a week over a four-week period and explore the vegetable of the month during a Sapere gathering once a week (see Sapere Menu 1). By introducing children to new foods, they get practice in enjoying healthy, varied meals.

Meal Practices Review Nutrition Now's ten research-based meal tips and watch the videos to create a shared understanding of the content among the staff. Discuss and decide how many and which of Nutrition Now's research-based meal tips you will work on at a time. Align and clarify the goals for the meals.

Parent Collaboration Check out Nutrition Now's tips for parent collaboration, and discuss how you will contribute to visible and open communication about food and meals.

Good luck,

Best regards, the researchers at Nutrition Now

Second Newsletter - (Week 44)

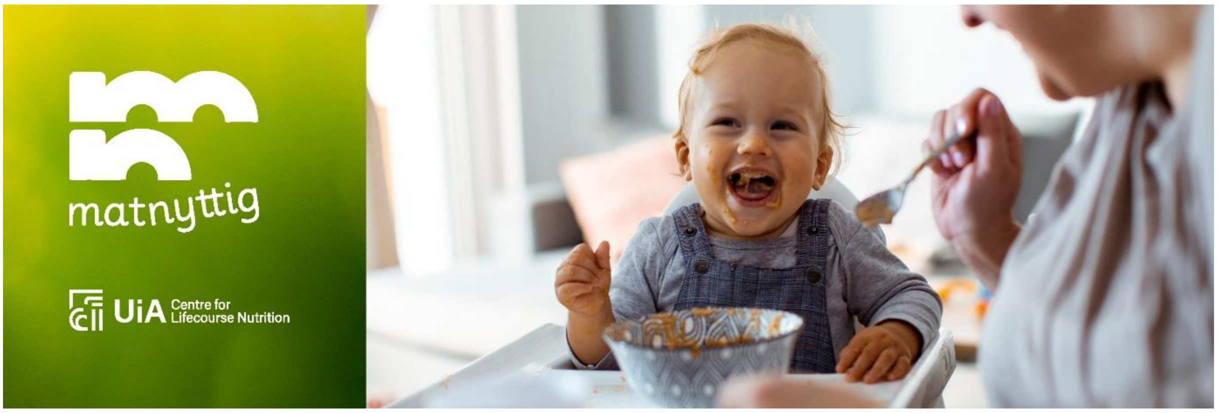

It's Almost Time for a New Menu and New Focus Vegetable

Check out the next menu proposal to see what the children will be served two days a week over the next four-week period. Select Monthly Menu 2 from the list, inform your colleagues about the menu change, and ensure a shopping list is prepared in good time. Also, check out the new focus vegetable and Sapere Menu 2.

Meal Practices and Leadership Your leadership is crucial for the implementation of Nutrition Now. Reflect on each staff member's skills and resources, adjust and organize yourselves as best as possible. We encourage evaluating the department's meal practices. Align and clarify the goals for the meals based on Nutrition Now's ten research-based meal tips and videos. Discuss how you can support and encourage each other to achieve the desired meal practices in everyday life. Set aside time during department meetings to establish expectations for each person's tasks and the collaboration needed to develop the department's food and meal practices. Provide feedback to each other and offer help when needed. Change and development processes can be more demanding at certain phases, so feel free to discuss with the manager.

Parent Collaboration Parent collaboration focusing on food and meals is vital for children's well-being and development. Feel free to get ideas from Nutrition Now's website.

The third menu starts in week 2, so remember to prepare for purchases before the Christmas break. We thank you for reading our newsletter and sharing our interest and joy in giving children a great start with healthy food.

Keep up the good work!

Best regards, the researchers at Nutrition Now

Third Newsletter (Week 1 - 2023)

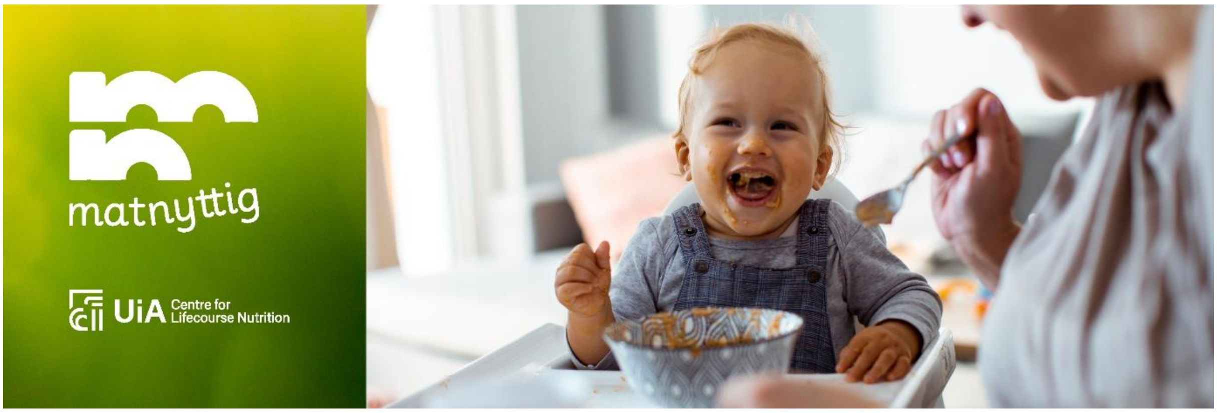

Continue the great work you are doing to give children a good start with food for life.

New Menu and New Focus Vegetable Take a look at Monthly Menu 3 and Sapere Menu 3 and find the recipes for the next four weeks. Inform your colleagues about the menu change and ensure a shopping list is prepared on time. Feel free to discuss sensory experiences during meals; it creates a connection between gathering moments and meals. Your leadership is crucial for the implementation of Nutrition Now. Contribute to a shared understanding of the work process, tasks, and the goals you are striving to achieve.

Learn from your experiences with meal practices and evaluate yourselves:

1. What are our goals for our mealtime practices?
2. Which mealtime educational advice are we using, and how are they used?
3. Continuation: Which mealtime educational advice will we apply going forward, and how will they be visible in practice?
4. Discuss how you can support and encourage each other to achieve desired practices in daily life.
5. Evaluate how reflection and discussion during department meetings contribute to collective learning and accountability – what are we doing to maintain/create a positive climate and perspective?

You can also find these tips described here (link to website).

We thank you for reading our newsletter and sharing our interest in further developing food and meal practices in daycare.

Best regards, the researchers at Nutrition Now

Fourth newsletter (week 4)

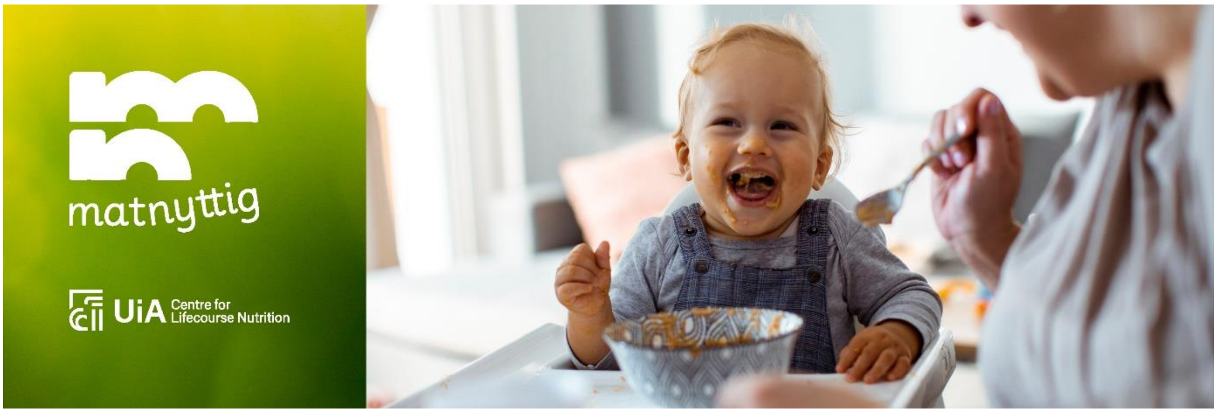

Continue the good work you are doing to provide children with a good food start for life

Halfway through the project period – it's time for the fourth menu and new focus vegetable

It's time to take a look at the monthly menu proposal 4, recipes to be prepared and served twice a week for the next four-week period. Also, check out Sapere menu 4 and find out what the new focus vegetable is in the Sapere session, this way children get repeated opportunities to learn to like new food. Inform your colleagues about the menu change and new focus vegetable and ensure that the shopping list is prepared on time.

Evaluate how 'Nutrition Now' has been working so far

In recent months, you and the staff in your department have gained a lot of experience in implementing the 'Nutrition Now' components, and thus learned many things. It is highly likely that some of your efforts have been noticed by parents and other staff members in the kindergarten.

Tip! Set aside time in department meetings to discuss perceived improvements or changes you believe are in the right direction. For example, you can start with visible changes in children, feedback from parents, or other colleagues in the kindergarten. The goal is to discuss each person's experience so that this becomes a shared learning and motivation for further development of the department's health-promoting food and mealtime practices.

Best regards, the researchers at Nutrition Now

Food and meals are health and pedagogy!

'Nutrition Now' contributes to realizing the framework's intentions for the kindergarten's work with food and meals. Reflection and collaboration within the department will contribute to both individual and collective learning, which will have a positive impact on the quality of the department's health-promoting and educational meal practices.

Fifth newsletter (week 8)

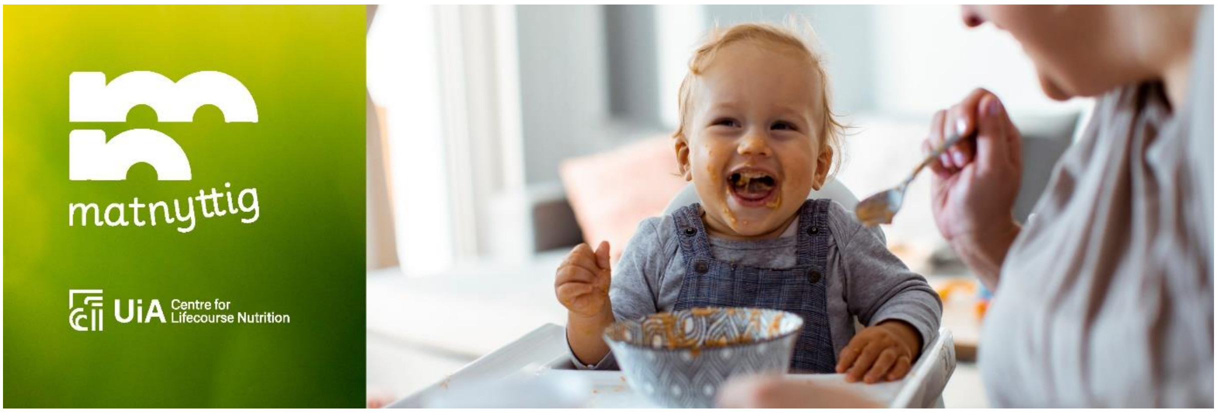

Continue with the good work you are doing to provide children with a good food start for life

Fifth menu and new focus vegetable

Choose monthly menu 5, inform your colleagues about the menu change, and ensure that the shopping list is prepared on time. Serve menu 5 twice a week for the next four-week period. Also introduce a new focus vegetable in the Sapere session and review Sapere menu 5. This way, children get repeated opportunities to learn to like new food.

Read together and discuss, a way to stimulate professional development

Professional development thrives in a professional community where, for example, you as a kindergarten teacher share and receive ideas and knowledge, evaluate, and further develop what seems important for the development of the kindergarten's health-promoting and educational food and mealtime practices. In recent years, food and health have been strengthened in the framework plan. In the Nutrition Now online resource, you can read about how the department's work with Nutrition Now aligns with the Framework Plan and the expectations and requirements of the Health Directorate.

Tip! For "knowledge work" in the staff group, you can, for example, ask your colleagues to read about how Nutrition Now's four components contribute to fulfilling the Framework Plan and the Health Directorate's expectations and requirements. Before a department meeting, ask your colleagues to read about, for example, cooking and how serving hot meals can be linked to the Framework Plan, discuss how you understand the text, evaluate, and decide how you can further develop what seems important for the development of the kindergarten's health-promoting and educational food and mealtime practices.

Feel free to split the reading into four smaller parts, one component for each meeting:

- Cooking (link)
- Sapere method (link)
- Mealtime practices (link)
- Parent collaboration (link)

A culture of systematic and developmental evaluation is central to the development of educational practices. This way, you can assess and reflect on whether the kindergarten's

mealtime practices are moving towards the goals you have and aligning with the Framework Plan.

Best regards, the researchers at Nutrition Now

Final newsletter (week 13)

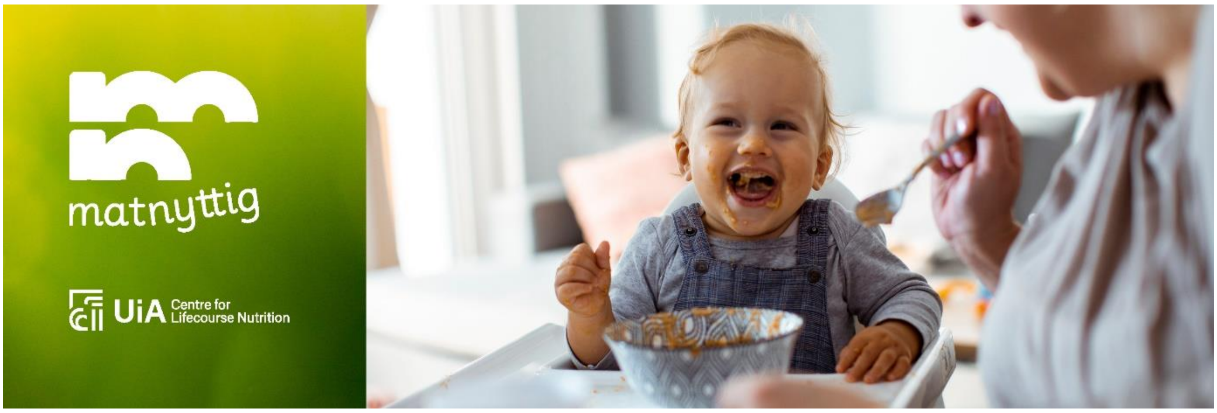

Well done!

Please continue with the good work you are doing and prevent children from becoming picky eaters. The more types of food and dishes a child has tasted before the age of two, the easier it will be to introduce new food in the preschool department. Set aside time in department meetings to discuss how you will continue and utilize the components of Nutrition Now to make it a lasting part of the department's practices.

Continue the good progress

• See our tips on the cooking page: Inspired to continue introducing children to new foods after the project period?

• See our tips on the Sapere session page: Inspired to continue with Sapere sessions after the project period?

• Continue to develop the department's health-promoting, mealtime educational practices.

• Continue to take initiatives for parent information and dialogue – feel free to review the video again, are there any tips and ideas you haven't tried or something you want to further develop?

We thank you for reading our newsletter, and especially for implementing the Nutrition Now project! Thank you also for sharing our commitment to giving children a healthy start with food for life.

Best regards, the researchers at Nutrition Now
