# Supplementary material for: Implementation strategies: lessons learned during an e-learning intervention to improve dietary behaviors and feeding practices in early childhood education and care
Source: BMC Nutr. 2025 Jan 13;11:7. doi: 10.1186/s40795-024-00990-3 (PMC11726949; doi:10.1186/s40795-024-00990-3)
Supplement: Supplementary file 4 — Supplementary Material 4: Supplementary Table 1. Interview guides. [file 40795_2024_990_MOESM4_ESM.pdf]

Supplementary Table 1 – Interview questions asked at each interview round

Question number	Week 41<br>**Planning and Information**	Week 45<br>**Newsletters, Leadership, and Core Components**	Week 48<br>**Team Meeting, Evaluation, and Leadership**	Week 2<br>**Getting Started After Christmas and Self-Evaluation**	Week 5<br>**Changes Related to Children and Parents**	Week 9<br>**Professional Development Among Staff**	Week 12<br>**How is mealtime practice aligned with the curriculum**	Week 15<br>**Further - professional development and use of *Nutrition Now***
1	As the team leader*, you have received information from us and the ECEC** manager, from us via email and the website. Has the information been sufficient to commence planning?	Since last time, you have received newsletters discussing initiating development processes among the staff. Was this helpful to you as a team leader?	There are four components in *Nutrition Now*. Can you tell me about how you work with these?<br><br>- Monthly menu<br>- Food sensory education sessions<br>- Pedagogical mealtime practices<br>- Parental cooperation	Have you resumed *Nutrition Now* activities after the Christmas break?	Have there been any changes among the children since you started with *Nutrition Now*?	*Did anyone from your* ECEC *participate in the team meeting/online meeting in week 7? Was it useful? If so, do you have any advice for us regarding future meetings?*	Before *Nutrition Now,* we conducted "a small survey" among ECEC staff. It emerged that there was a desire for a clear connection between *Nutrition Now* and the curriculum. At the bottom (on the page) of each of the four components on the *Nutrition Now* e-learning resource, the theme is linked to the curriculum. Have you noticed this?	*Nutrition Now* is a way to systematically work with food and meals in your ECEC. Do your ECEC plan to continue using *Nutrition Now* next fall?
2	If yes, inquire about what was good.<br>If no, ask what could have been helpful for you?	If not, what did you miss (in the newsletter)? (related to food and mealtime practices)	Did anyone from your ECEC participate in the team meeting/online meeting last week? (The reason we have these meetings is because we believe they are helpful for the implementation.) Do you have any feedback on the meeting?	During the first week of January, we sent an e-mail and a newsletter. Have you had time to look at them?	What about the parents? Have you received feedback from parents about any changes in their child's habits since you started with *Nutrition Now*?	Have there been any changes during mealtimes among the staff since you started with *Nutrition Now*? If so, which changes?	If yes, could you elaborate on that? If it could have been even better, is there anything we should have added or done differently?	Having completed *Nutrition Now*, is there any information from us that would make it easier for you to continue using *Nutrition Now*?
3	Is there anything in the e-learning resource that is difficult to understand?	How do you experience your role as a team leader?	For those who did not participate, could we do something differently so that you might be able to join the next meeting in week 7?	Do you feel that you are in a process where you evaluate and develop food- and mealtime competence in the department?	How do you experience your role as a team leader?	We provide tips through newsletters and team meetings, such as how to conduct evaluations to increase competence within the staff group. How well do these tips work?	Would it have been easier to use if there was a short video about it in the newsletters/website?	We're wondering about the newsletters: Do you think you would use the website more actively if the newsletters contained short videos guiding you to the right section on the e-learning resource, for example, about further use of *Nutrition Now* in the upcoming fall?
4	In *Nutrition Now*, there are four core components. You can find an overview of these on the website, and they are identical to the headings on the page. They are called:<br><br>- Monthly menu<br>- Gathering time<br>- Mealtime practices<br>- Parent collaboration<br><br>Do you understand these?<br>Do you think your colleagues have understood these?<br><br>If no: Have you thought about what you can do to help your colleagues gain a clear	Is there anything else you would like to add in conclusion?	How do you experience your role as a team leader?	For example, have you set aside regular times for evaluations, or have they been conducted sporadically?	Is there any type of support or support material you feel is lacking in your role as a team leader?	Is there anything else you would like to add in conclusion?	Is there anything else you would like to add in conclusion?	This is the last phone call. Is there anything important you would like to address?

	understanding of these (four components)?							
5	How do you experience your role as a team leader?		Is there anything else you would like to add in conclusion?	Is there anything else you would like to add in conclusion?	Is there anything else you would like to add in conclusion?			
6	What would be helpful for you as a team leader?							
7	Is there anything else you would like to add in conclusion?							

*team leader = champion ECEC**= Early childhood education and care
